# Unravelling the intricacies between gaming motivations and internet gaming disorder symptoms in adolescents: a network analysis of 2-year healthy and deteriorating transition profiles

**DOI:** 10.1186/s13034-023-00671-2

**Published:** 2023-10-21

**Authors:** Shiyun Chen, Shimin Zhu

**Affiliations:** Department of Applied Social Sciences, The Hong Kong Polytechnic University, Hung Hom, Kowloon, Hong Kong, Hong Kong SAR China

**Keywords:** Internet gaming disorder, Gaming motivation, Network analysis, Latent profile analysis, Adolescents

## Abstract

**Background:**

The increasing prevalence of internet gaming disorder (IGD) among adolescents has become a global concern, while gaming plays a large role in many adolescents’ lives. While prior research emphasised the significance of investigating IGD through an etiological lens, the interconnections between diverse gaming motivations and IGD symptoms in adolescents remain inadequately understood. This study explored the associations between distinct gaming motivations and IGD symptoms by conducting network analysis in adolescents.

**Methods:**

Data from a two-year longitudinal school-based survey (N = 2148) was utilized. LPA was used to offer a straightforward and interpretable solution for identifying adolescents with two-year healthy and deteriorating transitions of IGD profiles. Subsequently, we conducted a network analysis to explore and compare the associations between gaming motivations and IGD symptoms in adolescents with two-year healthy transition profiles and deteriorating transition profiles.

**Results:**

Three profiles were delineated: ‘low IGD’ (T1: n = 808, 37.62%; T2: n = 731; 34.03%), ‘middle IGD’ (T1: n = 1063, 49.49%; T2: n = 1103, 51.35%), and ‘severe IGD’ (T1: n = 277, 12.89%; T2: n = 314, 14.62%), classifying adolescents with healthy transitions and those with deteriorating transitions. Three gaming motivations (i.e., ‘Daily entertainment’, ‘I am good at it’, and ‘Improvement of ability and mindset’) were identified as protective motivations. Two others, ‘Enjoy being in the gaming world’ and ‘Preoccupation’, were identified as bridge nodes in adolescents with deteriorating transitions. Three core nodes (i.e., ‘Sense of victory’, ‘Enjoy being in the gaming world’, and ‘Sense of achievement’) were identified in both adolescents with healthy profile transitions and deteriorating transitions.

**Conclusion:**

Findings suggest that not all gaming motivations contribute to the development of IGD in adolescents. Adolescents with deteriorating transitions showed specific gaming motivation and IGD symptom that is closely connected. Future interventions should consider corresponding gaming motivation when addressing IGD among adolescents.

**Supplementary Information:**

The online version contains supplementary material available at 10.1186/s13034-023-00671-2

## Background

Internet gaming has become an integral part of many adolescents’ daily lives, raising concerns about its potential impact on their development. Internet games for leisure were highly associated with benefits such as relieving negative emotions and improving well-being [17]. However, previous research has found potential negative consequences of excessive gaming on adolescents’ psychological and social functioning, such as sleeping patterns [8], physical health [15], and mental health [40]. The fifth edition of the Diagnostic and Statistical Manual of Mental Disorders (DSM-5) refers to problematic internet behaviour as internet gaming disorder (IGD), which refers to the display of at least five of the following nine symptoms: (1) preoccupation, (2) withdrawal; (3) tolerance; (4) loss of control; (5) loss of non-gaming interests; (6) gaming despite psychosocial problems; (7) deception; (8) escaping/eliminating negative emotions; and (9) gaming despite negative consequences. Recent evidence suggested the prevalence of IGD was as high as 8.8% among adolescents [13]. Given the widespread popularity of gaming among adolescents, it is imperative to understand the etiological factors contributing to IGD in this population.

### The relationships between gaming motivations and IGD symptoms

One factor in understanding these etiologies is to view IGD from a motivational perspective, which is essential in avoiding overdiagnosis and pathologizing gaming behaviors [7, 49]. Drawing on Bartles’ grounded theory, Yee [50] developed a five-factor model of game motivation, which was further refined into the Motivation for Online Gaming Questionnaire (MPOGQ) after analysing the preferences of MMORPG players. This hierarchical model covers three dimensions: Achievement, Social, and Immersion. Within these dimensions are finer nuances, such as in the achievement dimension, where players find it rewarding to progress in the game; in the social dimension, where players find it rewarding to make deep connections with their peers; and in the immersion dimension, where players find it pleasurable to explore the game [1, 50]. However, while Yee's classification has been the cornerstone of numerous studies over the years, there is an emerging critique. Prior research suggested that the association between different gaming motivations and IGD symptoms may not consistently align, even when these motivations fall under the same categorical umbrella. For instance, Wang and Cheng [49] found that one type of immersion motivation, ‘interest in experiencing virtual worlds’, was negatively associated with IGD, which is the opposite of the effect of other immersion motivations. Heterogeneity for the classification of gaming motivation was also confirmed in another systematic review conducted by Bäcklund et al. [1] which examined the association between 26 factors in six gaming motivation models and IGD. This suggests a need for a more nuanced understanding of the potential recalibration of gaming motivation classifications in the context of IGD.

Furthermore, it has been argued that understanding this relationship from a symptomatological perspective can provide more accurate insights, as specific symptoms may manifest more prominently in relation to certain gaming motivations [49]. For instance, a study conducted in Hong Kong [30] demonstrated that certain motivations were significantly associated with specific IGD symptoms. Specifically, the study found that gamers' ‘social motivations’ were significantly correlated with IGD symptoms ‘preoccupations.’ These findings suggested that rather than adopting a single approach to studying IGD and its relationship to gaming motivations, it is important to dissect and analyse the unique interplay between IGD symptoms and various gaming motivations. This nuanced understanding has the potential to pave the way for more targeted interventions and preventative measures that target specific motivations that amplify certain IGD symptoms.

In the context of these challenging limitations, network analysis was considered one of the promising approaches to unravelling the intricacies between varying gaming motivations and IGD symptoms [16, 33]. According to network theory, the various gaming motivations and distinct IGD symptoms are presented as nodes within the network, enabling the visualisation of the roles played by different gaming motivations and the relationships between gaming motivations and various IGD symptoms [4, 5]. More importantly, network analysis facilitates the identification of core nodes for target intervention as well as bridge nodes (i.e., nodes of one variable that can activate the other variable), which offers a novel framework for intervention conceptualization [5]. Specifically, the results from network analysis help us identify potential intervention points within the network, whether by targeting specific symptoms or by altering the connections between IGD symptoms. This approach allows us to consider both individual symptoms and the broader network structure in informing effective prevention and intervention strategies for adolescents with IGD. Although previous studies have attempted to explore the relationship between gaming motivation and IGD symptoms through network analysis methods [16, 33], these studies have been conducted with adults rather than adolescents. Therefore, in the current study, we extended the investigation of network associations between gaming motivations and IGD symptoms in adolescent populations. We aim to address the following research problems by conducting network analysis: (1) to explore the network relationships between adolescents' game motivation and IGD symptoms and compare them, and (2) to identify the core and bridge nodes between game motivation and IGD symptoms.

### Consider the characteristics of IGD symptoms in adolescents

In the context of exploring the relationship between gaming motivations and IGD symptoms in adolescents, it becomes evident that we must grapple with two paramount considerations: the heterogeneity of IGD symptoms in the adolescent population [43, 44], and the dynamic nature throughout adolescent development [27, 47]. Given the heterogeneity of adolescents’ gaming behaviours [43, 44], using a threshold-based approach to delineate groups with and without IGD may not be accurate [43, 44]. For instance, Faulkner et al. [12] found that four profiles in terms of severity were shown based on the problematic gaming behaviours of 3338 high school students—namely, ‘normative’, ‘low’, ‘high’, and ‘severe’, rather than just grouping those with or without problematic gaming behaviours. Thus, a person-centred approach, ‘used to identify the dynamics of subgroups that emerge in a sample based on a selected set of variables, which is applicable to investigating research questions and hypotheses that aim to categorize subjects into common subgroups based on substantive variables’ [22], is an ideal approach to investigate potential subgroups based on the IGD symptoms in adolescents. Latent profile Analysis (LPA), one of the person-centered methods that focuses on identifying potential subgroups in a population based on a set of variables [22, 28], helps us to identify subgroups of adolescents based on the different IGD symptoms [42]. Prior to exploring gaming motivations and IGD symptoms, it is important to understand how adolescents should be grouped in terms of their IGD symptoms to be able to untangle the similarities and differences in how the two are directly related to each other in different subgroups of adolescents.

Moreover, measuring IGD in adolescents through cross-sectional data may not capture essential changes in adolescents. Since adolescence is a period characterised by significant physical and psychological changes, the severity of IGD symptoms in adolescents may fluctuate over time. Previous research found that only about one-third of addicted adolescent gamers exhibited stable levels of IGD over time, while the severity of gaming symptoms decreased over a 2-year period in most addicted adolescents [27, 47]. Thus, taking the developmental nature of adolescence into consideration, the current study aims to identify changes in heterogeneous IGD profiles of adolescents and group the adolescents in two-year healthy profile transition and deteriorating transition groups by using longitudinal data. In selecting our analytic approach, we carefully weighed the advantages and limitations of both LPA and Longitudinal Latent Profile Analysis (LLPA). While LLPA excels in modelling transitions between latent profiles over time [29], our primary research aims centred on characterizing these profiles at specific time points and identifying adolescents with two-year healthy transitions and deteriorating transitions of IGD profiles. In contrast, LPA provides more comprehensive information about adolescents in different profiles. Therefore, we chose to conduct the LPA at both time points and compare the transitions in adolescents' profiles. This approach offered a more straightforward and more interpretable solution aligned with our research goals.

### 2, 3 The current study

While most of the prior studies have explored the relationship between gaming motivation and IGD symptoms, the interaction between specific gaming motivations and different IGD symptoms remains ambiguous due to limitations in the lack of longitudinal data and analytic methods. The few studies that have used network analysis methods to fill this gap have not been conducted in adolescent populations, and thus the characteristics of the adolescent period have not been adequately considered. Latent profile analysis (LPA) was used to investigate subgroups of IGD in adolescents. Considering the developmental nature of adolescents, once we determined the subgroups throughout LPA in two time points, we used longitudinal data to identify changes in heterogeneous IGD profiles of adolescents and grouped the adolescents with two-year healthy transition and deteriorating transition of IGD profiles. Research questions are delineated below:

Research question 1: How are healthy and deteriorating transition profiles identified in adolescents via latent profile analysis across two time points?

Research question 2: What are the network relationships between gaming motivations and IGD symptoms in adolescents with two-year healthy transition and deteriorating transition of IGD profiles?

Research question 3: What distinctions and commonalities can be observed in the core and bridge nodes within the network of gaming motivations and IGD symptoms for adolescents with two-year healthy transition and deteriorating transition?

Given the exploratory nature of the three research questions presented, it becomes challenging to delineate concrete hypotheses and predictions.

## Methods

### Participants and procedures

The present study was a two-wave, longitudinal, school-based survey conducted over a two-year period, with data collected in 2021 (T1) and 2022 (T2). Ethical approval was obtained from the human subjects ethics subcommittee of the Hong Kong Polytechnic University (Ref. HSEARS20210414004-01). Invitations to participate were extended to 23 primary and secondary schools in Hong Kong, with recruitment concluding after four primary schools and 11 secondary schools agreed to participate. Consent was obtained from parents and respondents before the survey. Students were assured that their participation was voluntary and that they could withdraw at any time. Trained research assistants facilitated the survey administration in the classroom. Upon completion of each survey, participants were provided with a piece of stationery valued at approximately US$5.

A total of 3319 participants (attrition rate: 22.56%) completed both surveys at T1 and T2. The independent t-test and one-way ANOVA test found no statistically significant differences in gender (*p* = 0.352) or nationality (*p* = 0.183) between the final sample and the loss sample. Before proceeding with data analysis, participants who failed to provide valid data on any measures were excluded to ensure data quality. Ultimately, the final sample included 2148 participants, with a mean age of 13.95 (SD = 1.56), comprising 1119 boys (52.1%) and 1029 girls (47.9%). The demographic information about participants is shown in Table 1. In the present study, our participants were drawn from the general adolescent population with the aim of capturing a wide range of gaming behaviors. It is important to emphasize that this study did not target only adolescents with significant IGD behaviors. Rather, our goal was to use an integrative approach to better understand the nuances of gaming motivation and its association with IGD symptoms in a broader group of adolescents.

**Table 1:** Description of demographic information at T1 (N = 2148)

Variables	*N* (%)
Gender	
Male	52.1
Female	47.9
Age	
10–12	11.0
13–15	70.8
16–18	19.2
Garde	
Primary 4	5.4
Primary 5	5.8
Secondary 1	31.3
Secondary 2	23.6
Secondary 3	19.4
Secondary 4	14.5
Nationality	
Chinese	98.4
Non-Chinese	1.6
Father educational level	
Primary school qualification	4.5
Secondary Schools	47.4
University or higher	17.4
Unknown	29.3
Mother educational level	
Primary school qualification	7.4
Secondary Schools	49.1
University or higher	16.2
Unknown	25.9

### Assessments

#### Internet gaming disorder

The Chinese version of the 7-item Gaming Addiction Scale (GAS-7), developed based on the diagnostic criteria for IGD in the DSM-5, was used to measure internet gaming disorder symptoms at T1 and T2 [32, 51]. The GAS-7 employed a 6-point Likert scale ranging from 1 (‘strongly disagree’) to 6 (‘strongly agree’). Each item on the scale independently measured one symptom of IGD, resulting in a total of seven symptoms. The Cronbach’s alpha of this measurement was 0.84 at T1 and 0.86 at T2 in this study.

#### Gaming motivations

The adapted version of the Motives for Online Gaming Questionnaire (MOGQ) was used to assess gaming motivations in this study [7]. The original 27-item questionnaire consisted seven uncorrected domain-specific factors (i.e., Escape, Coping, Fantasy, Skill Development, Recreation, Competition, and Social) with three to four items in one factor. As schools could only arrange a limited time for classroom surveys in the brief the school resumption during the COVID-19 pandemic, we abridged the scale into 12 items, to ensure sufficient time for participants to read and answer the survey carefully and revise the original 5-point Likert into binary response format. We conducted a pilot study and sought feedback about the understandability and relevancy of all items from two school social workers and eight school students in participating schools. Based on the feedback collected, we picked one (maximumly two items) most understandable and representative of each factor. Furthermore, three additional items—namely ‘Exciting and fun’, ‘I am good at it’, and ‘Sense of achievement’—were included based on students’ responses to an open-ended question regarding their gaming motivations. The final measure consisted of 12 items, and each represents one type of gaming motivations in our analysis. A binary response format was employed (0 = No; 1 = Yes) in this measurement. The total score of all items was not utilised in the subsequent analysis of gaming motivations, so no reliability analysis of the scale was performed in this study.

### Analyses plan

#### Latent profile analysis (LPA)

LPA with the maximum likelihood estimator was conducted using Mplus 8.0 [35]. LPA models were separately estimated for two time points, employing the 7 IGD symptoms as profile indicators. Solutions ranging from 1 to 5 latent profiles were examined at each time point. The best solution was determined based on the following criteria [36]: (1) lower values of the Akaike information criterion (AIC), Bayesian information criterion (BIC), and sample-size-adjusted Bayesian information criterion (aBIC); (2) higher entropy (higher than 0.80); and (3) a significant Lo-Mendel-Rubin likelihood ratio test (LMR) and the bootstrap-based likelihood ratio test (BLRT). Furthermore, the minimum potential subgroup size was required to be at least 5% of the total population. Based on the identified profiles at T1 and T2, adolescents were classified as two-year healthy profile transition and deteriorating transitions group.

#### Network analysis

Rstudio version 4.2.2 was used for network analysis. Missing cases were handled with listwise deletion as the chosen estimation algorithm does not allow for missing data [6].

Initially, the network was estimated using a mixed graphical model (MGM) in the R package ‘mgm’ [18], separately for adolescents with two-year healthy profile transition and deteriorating transitions of IGD profiles. In the networks, each gaming motivation and IGD symptom was considered a ‘node’, and ‘edges’ between nodes were understood as partial correlations. To avoid false positive findings, MGM uses the minimum absolute shrinkage and selection operator [45], following an extended Bayesian information criterion (EBIC). The R package ‘qgraph’ [10] was used to visualise the network structure. Green edges illustrate positive associations, and red edges represent negative associations. Wider and more saturated edges mean stronger associations.

Secondly, we examined the centrality of expected influence for each item [19], which reflects the total level of involvement of a node in the network. The non-parametric bootstrap for 1000 times from the ‘bootnet’ package was conducted to estimate whether there were significant differences in expected influence between each node and other nodes, thus identifying the core nodes with significantly higher expected influence in the networks. Since expected influence only addresses the relative importance of the nodes, the predictability of each node was also estimated. Predictability quantifies the extent to which a given node can be predicted by all other nodes in the network [24], and it is an absolute measure of interconnectedness as it provides us with the variance (*R*^2^) of a node being explained by all its neighbours. If a node exhibits a high level of expected influence and predictability, it supports the interpretation of its importance in the network. Thirdly, the bridge function from the R package ‘networktools’ was used to identify the bridge nodes. One-step bridge expected influence was calculated, which represented the sum of the edge weights connecting a given node to all nodes in the other variable, to identify important nodes that serve as bridges between variables.

Finally, to assess the stability of the networks and the accuracy of edges, the non-parametric 1000-iterations bootstrap was conducted [20]. The width of the confidence intervals (CIs) provided an indication of the edges’ stability, with narrower CIs suggesting greater trustworthiness. A case-drop bootstrap was conducted to examine the accuracy of centrality by assessing the correlation between estimated centrality in the original and subset samples. Stability was quantified using correlation stability coefficients (CS-coefficients), which were expected to exceed 0.5 but not fall below 0.25 [9].

## Results

The descriptive findings of gaming motivations and IGD symptoms during T1 and T2 are provided in Table 2. It’s worth noting that our participants predominantly displayed mild to moderate IGD symptoms. The average scores of IGD symptoms, as presented in Table 2, reveal that most symptoms scored below 3, except for tolerance.

**Table 2:** Descriptive analysis of gaming motivations and IGD symptoms across two time points (N = 2148)

Items	T1		T2	
Gaming motivations	None (%)	Yes (%)	None (%)	Yes (%)
M1: Playing with friends	28.2	71.8	26.9	73.1
M2: Meeting new friends	68.9	31.1	69.3	30.7
M3: Sense of victory	54.6	45.4	54.7	45.3
M4: Forgetting to be upset	42.6	57.4	42.8	57.2
M5: Daily entertainment	24.3	75.7	24.3	75.7
M6: Exciting and fun	55.2	44.8	53.2	46.8
M7: I am good at it	78.4	21.6	76.4	23.6
M8: Like the character/story	67.7	32.3	61.3	38.7
M9: Sense of achievement	62.2	37.8	54.1	45.9
M10: Coping with stress	57.3	42.7	56.7	43.3
M11: Improvement of ability and mindset	64.9	35.1	65.5	34.5
M12: Enjoy being in the gaming world	74.0	26.0	70.0	30.0

IGD symptoms	Mean	SD	Mean	SD
Preoccupation	3.00	1.23	3.02	1.22
Tolerance	3.55	1.37	3.60	1.34
Gaming despite harms	2.99	1.36	3.06	1.34
Loss of control	3.02	1.45	2.90	1.33
Withdrawal	2.49	1.34	2.49	1.26
Conflict due to gaming	2.88	1.49	2.98	1.47
Loss of non-gaming interests	2.49	1.33	2.58	1.30

### RQ 1. Determining the healthy and deteriorating transitions in adolescents based on profile transitions at two-time points

**Fig. 1:**
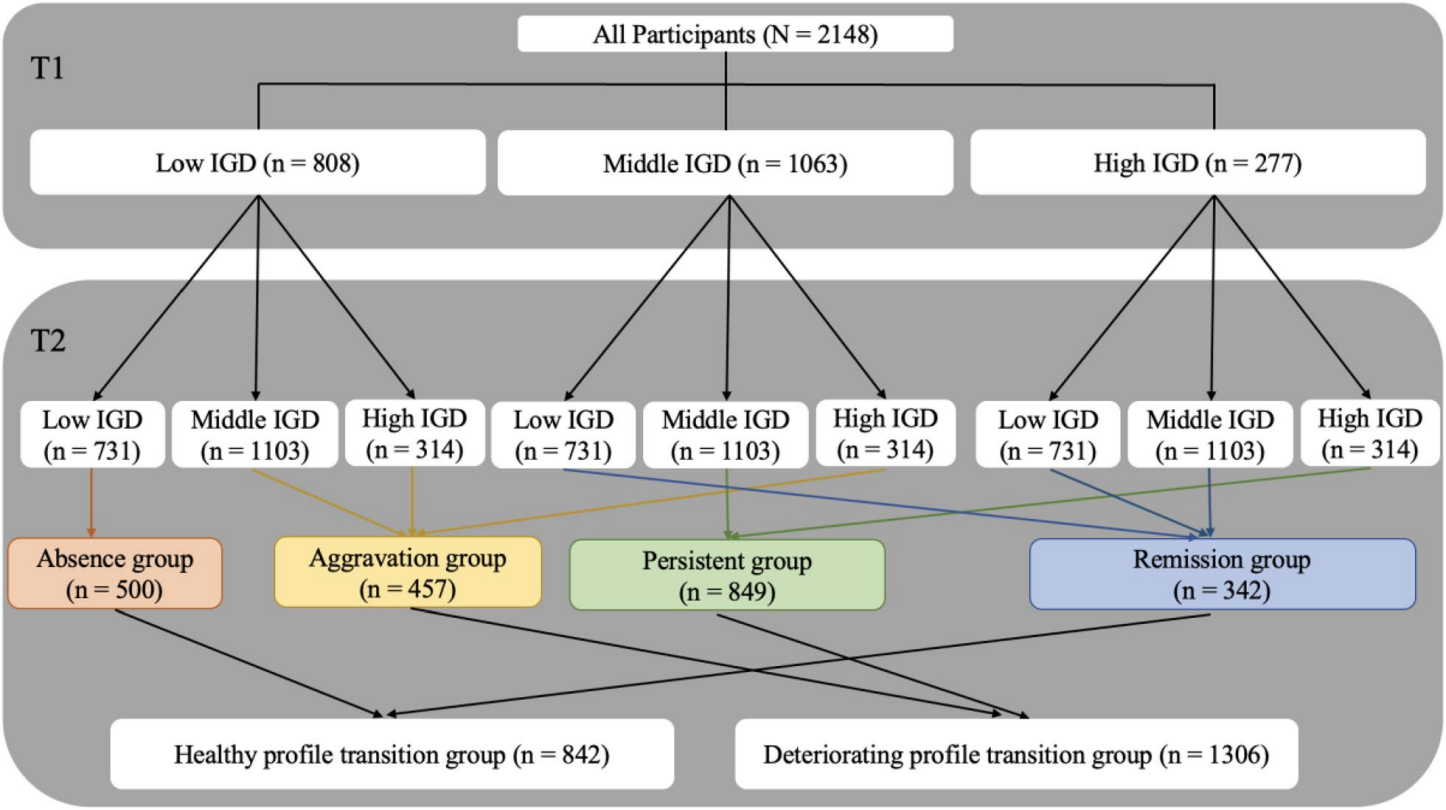
Flowchart of grouping participants based on the IGD profile transition at two-time points

The identification of health transition and deterioration transition groups for adolescents specifically includes three steps (see Fig. 1).

**Fig. 2:**
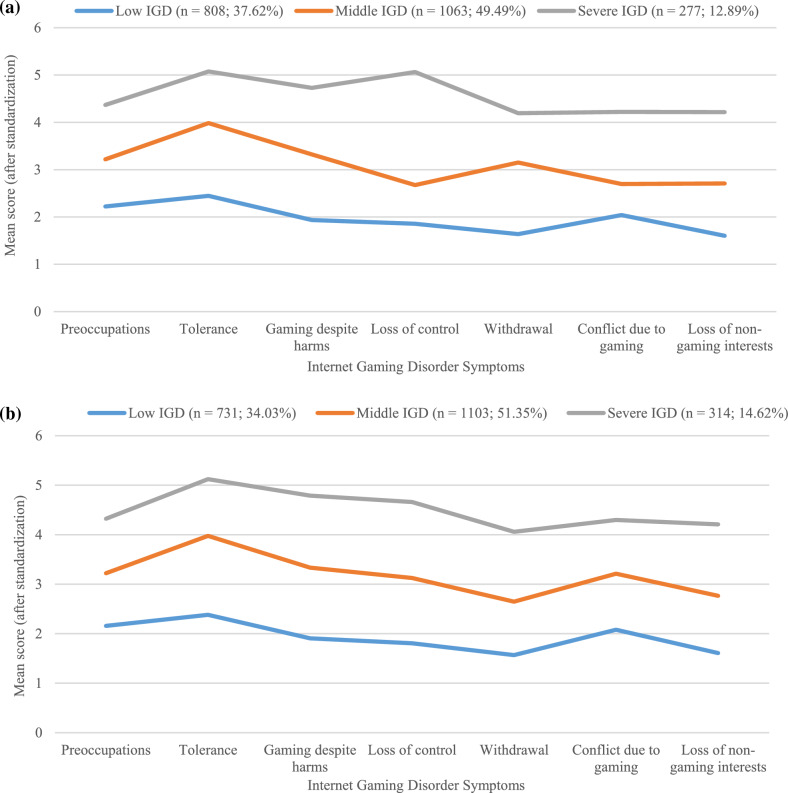
**a** Profile plot of three-profile model at T1. **b** Profile plot of three-profile model at T2

Initially, the findings consistently indicated that the 3-profile model was the most optimal representation of the data at both time points (the goodness of fit is shown in the Table 3). Profile 1, consisting of 37.62% (*n* = 808) of participants at T1 and 34.03% (*n* = 731) participants at T2, was designated as the ‘low IGD’ group. Profile 2, denoted as the ‘middle IGD’ group, encompassed 49.49% (*n* = 1063) of the sample at T1 and 51.35% (*n* = 1103) of the sample at T2. Profile 3, representing 12.89% (*n* = 277) of the sample at T1 and 14.62% (*n* = 314) of the sample at T2, was identified as the ‘severe IGD’ group. The characteristics of IGD symptoms among the three profiles at T1 and T2 were shown in Fig. 2a and b.

**Table 3:** Goodness of Fit for the LPA of the IGD symptoms at Two-time Points

Class	K	Log(L)	AIC	BIC	aBIC	Entropy	LMR	BLRT	Probability of each profile (%)
T1									
1	18	− 31,639.599	63,315.198	63,417.299	63,360.111	–	–	–	–
2	24	− 24,181.972	48,411.944	48,548.079	48,471.829	0.794	< 0.001	< 0.001	56.24/ 43.76
3	34	− 23,626.966	47,321.933	47,514.79	47,406.768	0.816	< 0.001	< 0.001	37.62/ 49.49/12.89
4	44	− 23,457.637	47,003.273	47,252.854	47,113.061	0.846	> 0.05	< 0.001	38.13/46.04/3.54/12.29
5	54	23,320.301	46,748.601	47,054.905	46,883.34	0.791	> 0.05	< 0.001	29.94/20.90/38.87/4.56/5.73
T2									
1	18	− 31,172.470	62,380.940	62,483.041	62,425.853	–	–	–	–
2	24	-23,516.520	47,081.04	47,217.175	47,140.924	0.805	< 0.001	< 0.001	52.65/47.35
3	34	− 22,876.323	45,820.646	46,013.504	45,905.482	0.822	< 0.01	< 0.001	34.03/51.35/14.62
4	44	− 22,658.253	45,404.507	45,654.09	45,514.294	0.780	< 0.01	< 0.001	5.73/40.50/24.02/29.75
5	54	− 22,465.18	45,038.36	45,344.664	45,173.10	0.812	< 0.05	< 0.001	24.53/3.45/39.62/26.91/5.49

*Log(L)* log likelihood, *K* number of estimated parameters, *AIC* akaike information criterion, *BIC* Bayesian information criterion, *aBIC* adjusted BIC, *LMR* Lo-Mendel-Rubin likelihood ratio test, *BLRT* Bootstrap likelihood ratio test

Secondly, according to the transitions of three profiles across the two time points, participants were allocated into four distinct groups: (1) Absence group (*n* = 500), comprising adolescents classified as the Low IGD group at both T1 and T2; (2) Aggravation group (*n* = 457), consisting of adolescents who were classified as the Low IGD group at T1 but who transitioned to either the Middle IGD or Severe IGD group at T2, or alternatively, who were initially classified as the Middle IGD group at T1 but who shifted to the Severe IGD group at T2; (3) Persistent group (*n* = 849), including adolescents consistently classified as either the Middle IGD or Severe IGD group at both T1 and T2; (4) Remission group (*n* = 342), consisting of adolescents who exhibited a decrease in IGD severity from the Severe IGD group at T1 to either the Middle IGD or Low IGD group at T2, or alternatively, who transitioned from the Middle IGD group at T1 to the Low IGD group at T2.

Thirdly, upon examining the profile transitions of above four groups, two predominant patterns emerged. Participants in the ‘absence’ and ‘remission’ profiles both indicated a trend of low or diminishing IGD scores over time. These were hence combined to form a singular ‘healthy profile transition group’ (*n* = 842). On the other hand, participants categorized under ‘aggravation’ and ‘persistent’ demonstrated either sustained or amplifying IGD scores, prompting their classification into a consolidated ‘deteriorating profile transition group’ (*n* = 1306).

### RQ 2. Network relationships between gaming motivations and IGD symptoms in adolescents with two-year healthy profile transitions and deteriorating transitions

**Fig. 3:**
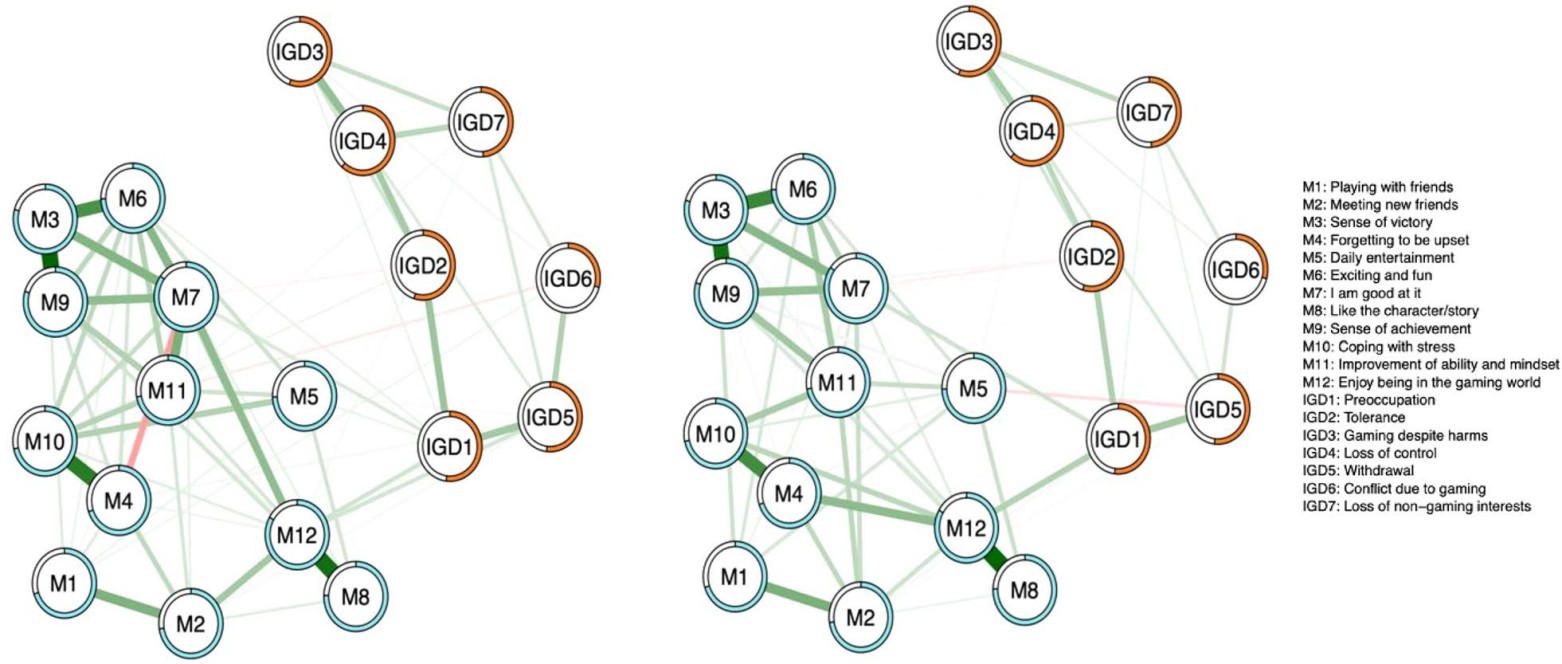
The network structure of healthy profile transition group (left, n = 842) and deteriorating profile transition group (right, n = 1306) at T1. Blue nodes indicate game motivation, and orange nodes indicate IGD symptoms. Thicker edges between nodes denote stronger associations. Green edges denote positive interconnections, red edges denote negative interconnections, and gray edges denote non-significant interconnections

Figure 3 presents networks of adolescents with healthy profile transitions and deteriorating transitions at T1. Both networks included 171 edges, with a total of 41 non-zero-weight edges (24.0%) in the healthy profile transition group and 52 non-zero-weight edges (30.4%) in the deteriorating profile transition group. The CS coefficient of edges was 0.71 and 0.75 in the healthy profile transition group and the deteriorating profile transition group, respectively (see Additional file 1: Fig. S1). Consistent negative associations were observed in both the healthy profile transition group and deteriorating profile transition group, including the edge between the motivation ‘Daily entertainment (M5)’ and the symptom ‘Withdrawal (IGD5)’ as well as the edge between the motivation ‘Improvement of ability and mindset (M11)’ and the symptom ‘Loss of control (IGD4)’. In the healthy profile transition group, negative associations were revealed between the motivation ‘Improvement of ability and mindset’ and the symptom ‘Conflict due to gaming (IGD6)’, between the motivation ‘I am good at it (M7)’ and the symptom ‘Loss of non-gaming interests (IGD7)’, and between the motivations ‘Forgetting to be upset (M4)’ and ‘I am good at it (M7)’. In the deteriorating profile transition group, negative associations were observed between the motivation ‘Daily entertainment (M5)’ and the symptom ‘Loss of control (IGD4)’, between the motivation ‘I am good at it (M7)’ and the symptom ‘Tolerance (IGD2)’, between the motivation ‘Improvement of ability and mindset (M11)’ and the symptom ‘Loss of non-gaming interests (IGD7)’. Taken together, consistently protective gaming motivations were shown in both adolescents with two-year healthy transitions and deteriorating transitions of IGD profiles, including ‘Daily entertainment (M5)’, ‘I am good at it (M7)’, and ‘Improvement of ability and mindset (M11)’. Differently, a negative association between gaming motivations could be observed in adolescents with healthy profile transitions (i.e., ‘M4—Forgetting to be upset’ and ‘M7—I am good at it’).

### RQ 3. The core and bridge nodes in adolescents with two-year healthy profile transitions and deteriorating profile transitions

**Fig. 4:**
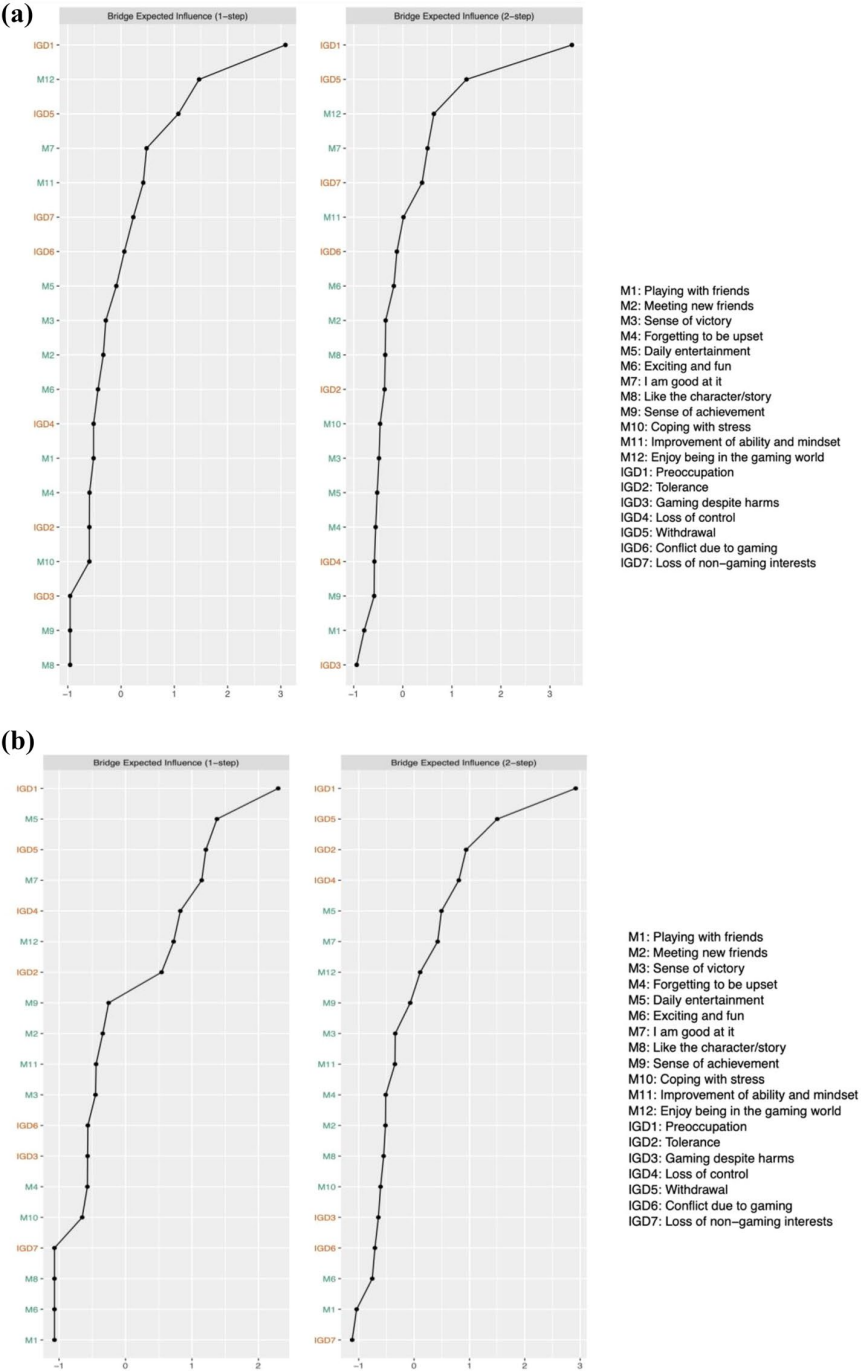
**a** Bridge expected influence of healthy profile transition group (left, n = 842). **b** Bridge expected influence of deteriorating profile transition group (right, n = 1306)

The bridge expected influence of the networks (see Fig. 4a and b) showed that in the healthy profile transition group, although the symptom ‘Preoccupation (IGD1)’ displayed the highest bridge expected influence, it failed to show a significantly higher value than most other nodes (see Additional file 1: Fig. S2). In the deteriorating profile transition group, the symptom ‘Preoccupation (IGD1)’ and the motivation ‘Enjoy being in the gaming world (M12)’ displayed significantly higher bridge expected influences compared to most other IGD symptoms. The CS-coefficients of bridge expected influence were 0.09 in the healthy profile transition group and 0.47 in the deteriorating profile transition group, which highlighted the need for caution when interpreting bridge nodes in the healthy profile transition group.

**Fig. 5:**
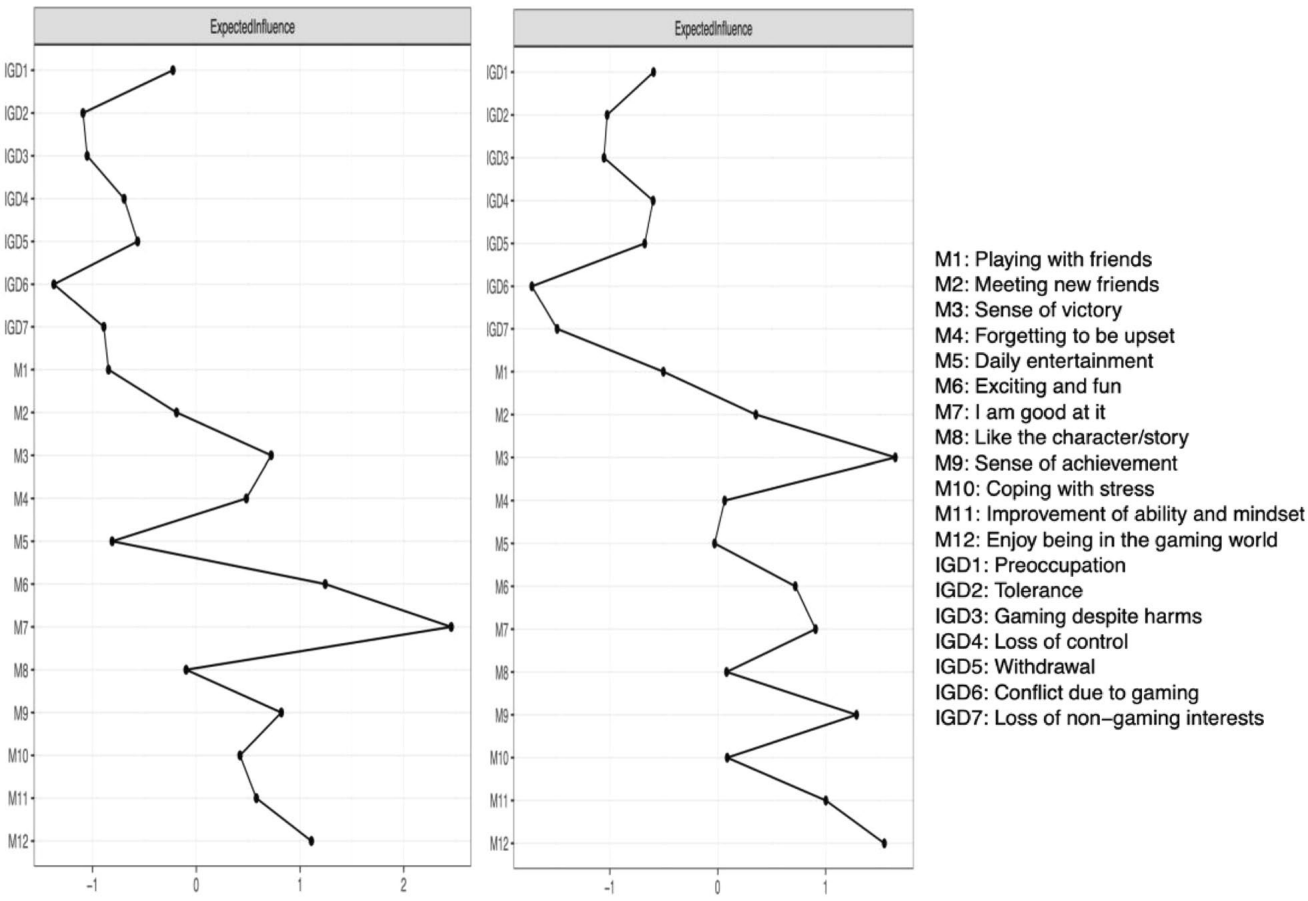
Expected influence of healthy profile transition group (left, n = 842) and deteriorating profile transition group (right, n = 1306)

The results of expected influence revealed that gaming motivations exhibited higher expected influence compared to IGD symptoms. In both the healthy profile transition and deteriorating profile transition groups, the motivations ‘Sense of victory (M3)’, ‘Enjoy being in the gaming world (M12)’, and ‘Sense of achievement (M9)’ demonstrated the highest expected influence (see Fig. 5), which showed significant differences from most other motivations based on the results of the bootstrap test (see Additional file 1: Fig. S3). Predictability estimations showed a high overall explanation of IGD symptoms by associated nodes in the healthy profile transition group (mean of 50%). In the deteriorating profile transition group, a low overall explanation of IGD symptoms by associated nodes was displayed (mean of 32%), which implies that IGD symptoms for adolescents with deteriorating profile transitions were determined by factors not included in the network. The CS-coefficients of expected influence were 0.57 in the healthy profile transition group and 0.53 in the deteriorating profile transition group, implying that the estimated expected influence was relatively stable.

## Discussion

Given the heterogeneity and developmental nature of adolescents’ gaming behavior, this study first identifies adolescents in the healthy profile transition group and deteriorating profile transition group by conducting LPA in two time points (RQ1). Next, the current study used network analysis to further our understanding of the interplay between varying gaming motivations and different IGD symptoms in adolescents with the healthy profile transitions and deteriorating profile transitions (RQ2) and identify the core nodes and bridge nodes in the network of gaming motivations and IGD symptoms (RQ3). Throughout the identification of bridge nodes, we have a clear picture of the most important gaming motivations that influence the development of IGD, and the most crucial IGD symptoms that reinforce the gaming motivations. Identifying the core nodes, which represent the primary and most influential nodes that hold significant sway over the entire network, is able to highlight the target of the intervention programmes.

The findings from the LPA suggest that three distinct IGD profiles (i.e., ‘Low IGD’, ‘Middle IGD’, and ‘Severe IGD’) occurred among adolescents at both time points. These findings, together with previous research, confirm the heterogeneity of IGD in the adolescent population [46]. Notably, only 39.2% of adolescents showed healthy profile transitions, while most of them (60.8%) displayed deteriorating profile transitions. These findings highlight that problematic gaming is not a transient issue for most adolescents [14] and that the severity of IGD should be considered in conjunction with its progression and evolving trends. Furthermore, the longitudinal design allowed us to capture the dynamic nature of IGD behaviours across two assessment points. By consolidating the profiles at two time-points, we were able to categorize the IGD features into four groups (i.e., Absence group, Aggravation group, Persistent group, and Remission group). To provide a practical perspective for targeting interventions and thus identifying overarching patterns associated with IGD profiles, we further divided into a healthy profile transition group and a deteriorating profile transition group by summarizing the characteristics of the four groups. While the detailed nuances of the four different profiles are invaluable, simplifying the distinction is imperative to gain actionable insights, especially in the intervention arena.

The findings from network analysis reveal that not all gaming motivations were risky for developing IGD symptoms. Specifically, three gaming motivations—‘Daily entertainment’, ‘I am good at it’, and ‘Improvement of ability and mindset’—showed negative associations with specific IGD symptoms in adolescents with two-year healthy profile transitions and deteriorating transitions of IGD profiles. These results further confirm the potential benefits of gaming for adolescents as an effective approach to relaxation and stress reduction [38, 41, 48]. Previous studies have been inconsistent in the relationships between such recreational motivations and IGD. For instance, the systematic review and meta-analysis by Bäcklund et al. [1] found that recreational motivations were significantly and positively associated with IGD. However, in one synthesis review, Király et al. [25] found that most studies concluded that individuals who play solely for entertainment purposes are very unlikely to develop IGD [2, 3, 26]. In this study, the findings support that recreational motives such as ‘Daily entertainment’ serve as protective motivations for IGD. Previous research suggested that low levels of gaming contribute to emotional stability [37] and reduce emotional disturbance in children [23]. This study substantiates the preventive potential of the appropriate use of leisure games in adolescents for maintaining a positive mental health state and for helping prevent problematic internet behaviour [34]. Moreover, the protective effects of the motivations ‘I am good at it’ and ‘Improvement of ability and mindset’ suggest that a moderate sense of competence derived from gaming can safeguard adolescents from excessive gameplay. Similar to previous studies, the psychological allure of gaming stems from its ability to generate feelings of competence, such as observable increases in skills and positive feedback, which fulfil the psychological need for challenge and efficacy [39]. The augmented sense of competence gained through gameplay fosters self-esteem and self-efficacy in adolescents, equipping them with coping mechanisms to effectively navigate real-life challenges and reducing the likelihood of IGD development [52]. Furthermore, this study found a negative association between certain gaming motivations (i.e., ‘Forgetting to be upset’ and ‘I am good at it) in adolescents with healthy profile transitions. This suggests a distinct difference between the motivations driving adolescents in the healthy profile transition group compared to those in the deteriorating profile transition group. Specifically, adolescents with healthy profile transitions may be driven by a specific, primary need to engage in gaming, whereas adolescents with deteriorating profile transitions may be driven by a combination of needs behind gaming motivations. However, since the relationship between gaming motivations is not the main objective of this research, future research could further examine these relationships.

We found that, in the deteriorating profile transition group, the gaming motivation ‘Enjoy being in the gaming world’ served as a pivotal bridge node leading to the emergence of IGD, while the symptom ‘preoccupation’ was identified as the bridge node reinforcing the gaming motivations. Specifically, ‘Enjoy being in the gaming world’ is the most crucial motivation that led to the development of IGD communities, while the symptom ‘preoccupation’ is the one that reinforces the gaming motivations. The gaming motivation ‘Enjoy being in the gaming world’ signifies adolescents’ fascination with an alternative world separate from reality, where they can assume an alternative self-concept. Self-concept encompasses how individuals perceive themselves based on attributes such as skills, abilities, appearance, and behaviours, encompassing the actual self, ideal self, and ought self [31]. Higgins posited that distress was experienced when there was a significant difference between a person’s actual self and ideal self and that such distress drove them to unify these self-perceptions [21]. Thus, gaming may serve as an avenue through which adolescents can reduce the discrepancy between their ideal and actual selves, primarily through strong identification with their game avatars. According to self-discrepancy theory, these individuals are driven to sustain this behaviour to maintain the reduction in self-discrepancy [21], which may consequently contribute to the development or perpetuation of IGD. Furthermore, the findings of this study suggest that in adolescents with deteriorating profile transitions, the IGD symptom ‘Preoccupation’ was perceived as a bridge node that further reinforced gaming motivations. This result implies that when adolescents with deteriorating profile transitions exhibit a preoccupation with gaming, they are more inclined to satisfy hidden needs, such as social needs and entertainment needs, by gaming.

The centrality of the networks underscores the significance of targeting gaming motivations in interventions aimed at preventing IGD symptoms, which is consistent with previous studies (e.g., [25]. Centrality quantifies the relative importance of gaming motivations within the network, indicating the influence or connectivity of gaming motivations to other nodes. The findings reveal that all gaming motivations hold higher expected influence indices compared to IGD symptoms, indicating that interventions focusing on modifying gaming motivations may yield greater efficacy than directly addressing IGD symptoms. The motivations ‘Enjoy being in the gaming world’, ‘Sense of victory’, and ‘Sense of achievement’ were considered core nodes, which showed higher expected influence compared to other gaming motivations. The motivation ‘Enjoy being in the gaming world’, served as a pivotal motivation not only in triggering IGD development but also in activating other gaming motivations. The motivation ‘Sense of victory’ as a core node suggested that being capable of gaining a sense of victory from games with a competitive component may be detrimental to adolescents, which further supported the negative effects of games’ competitive aspects relating to adolescent development, such as the inability to form healthy cooperative relationships with peers [11]. Such a negative impact may further reinforce other gaming motivations, such as ‘Meeting new friends’, thereby escalating reliance on gaming, and contributing to the development of IGD. The prominent role of the motivation ‘Sense of achievement’ highlighted the importance of addressing young individuals’ aspirations for real-life accomplishments and supporting them in cultivating achievements through avenues beyond gaming. Taken together, targeting gaming motivations in interventions to prevent IGD symptoms was crucial, particularly to address underlying needs driven by the motivations ‘Sense of victory’, ‘Enjoy being in the gaming world’, and ‘Sense of achievement’.

While this study shows the potential of the network approach in advancing our comprehension of gaming motivations and IGD symptoms in adolescents with two-year healthy profile transitions and deteriorating profile transitions of IGD profiles, there are some limitations. Firstly, our sample was largely representative of adolescents with mild to moderate IGD symptoms, making the findings potentially less applicable to clinical populations with severe IGD. Future research targeting clinical groups would be instrumental in drawing comprehensive conclusions regarding gaming motivations and IGD symptoms across the severity spectrum. The applicability of the assessment of gaming motivation would need to be adjusted if the sample consisted of pathological gamers. Secondly, the participants in the current study had a broad age range, which may vary in the relationship between gaming motivations and IGD symptoms. Future research can further examine the differences between different age groups. Thirdly, although we adopted attention-checking to monitor the survey quality, the self-reported measures may be susceptible to social-desirability bias. Future research endeavours could incorporate more ecologically valid methodologies, such as daily diary designs or screen time monitor apps, to further examine and validate the outcomes of this study. Fourthly, it's important to acknowledge that our study is limited by the absence of a standardized measurement method for gaming motivation, particularly tailored to adolescents. While we adapted the MOGQ scale and employed network analysis methods that consider each item to represent unique dimensions independently, we did not conduct traditional psychometric validations. This approach allowed us to focus on individual items rather than the entire scale but also meant that we couldn’t fully capture the effects of potential classifications within gaming motivations. We recognize this limitation and recommend that future research should include a more comprehensive psychometric validation of the adapted scale. Finally, the study did not employ a directional network analysis because of the primary research objective of this study, although we used LPA with longitudinal data to identify groups with IGD changes. Future investigations can employ cross-lagged network analysis methods to further enhance our comprehension of this issue.

## Conclusion

Gaming is a popular leisure activity and adolescents play games with different motives. Three protective gaming motivations (‘Daily entertainment’, ‘I am good at it’, and ‘Improvement of ability and mindset’) were identified in adolescents with healthy profile transitions and deteriorating profile transition. Bridge nodes—namely, the motivation ‘Enjoy being in the gaming world’ and the IGD symptom ‘Preoccupation’—were observed in adolescents with deteriorating profile transitions. The results of this study provide insights into intervention programmes to reduce IGD in adolescents, emphasising the importance of intervening from the perspective of motivation with a particular focus on three motivations: ‘Sense of victory’, ‘Enjoy being in the gaming world’, and ‘Sense of achievement’.

### Supplementary Information

**Additional file 1: Figure S1.** Stability estimations of edges using the case-drop bootstrapping method of healthy profile transition group and deteriorating profile transition group. **Figure S2.** The difference test results of bridge expected influence using the non-parametric bootstrapping method in healthy profile transition group and deteriorating profile transition group. **Figure S3.** The difference test results of expected influence using the non-parametric bootstrapping method in healthy profile transition group and deteriorating profile transition group.

## Data Availability

The datasets used and/or analysed during the current study are available from the corresponding author on reasonable request.

## Abbreviations

IGD: Internet gaming disorder
LPA: Latent profile analysis
DSM-5: Diagnostic and statistical manual of mental disorders-5
GAS-7: 7-Item gaming addiction scale
MOGQ: Motives for online gaming questionnaire
AIC: Akaike information criterion (AIC)
BIC: Bayesian information criterion
aBIC: Sample-size-adjusted Bayesian information criterion
LMR: Lo-Mendel-Rubin likelihood ratio test
BLRT: Bootstrap-based likelihood ratio test
MGM: Mixed graphical model (MGM)
EBIC: Extended Bayesian information criterion
CIs: Confidence intervals
CS- coefficients: Correlation stability coefficients
