# Supplementary material for: Unravelling the intricacies between gaming motivations and internet gaming disorder symptoms in adolescents: a network analysis of 2-year healthy and deteriorating transition profiles
Source: Child Adolesc Psychiatry Ment Health. 2023 Oct 21;17:122. doi: 10.1186/s13034-023-00671-2 (PMC10590022; doi:10.1186/s13034-023-00671-2)
Supplement: Supplementary file 1 — Additional file 1: Figure S1. Stability estimations of edges using the case-drop bootstrapping method of healthy profile transition group and deteriorating profile transition group. Figure S2. The difference test results of bridge expected influence using the non-parametric bootstrapping method in healthy profile transition group and deteriorating profile transition group. Figure S3. The difference test results of expected influence using the non-parametric bootstrapping method in healthy profile transition group and deteriorating profile transition group. [file 13034_2023_671_MOESM1_ESM.docx]

**Appendices**

**
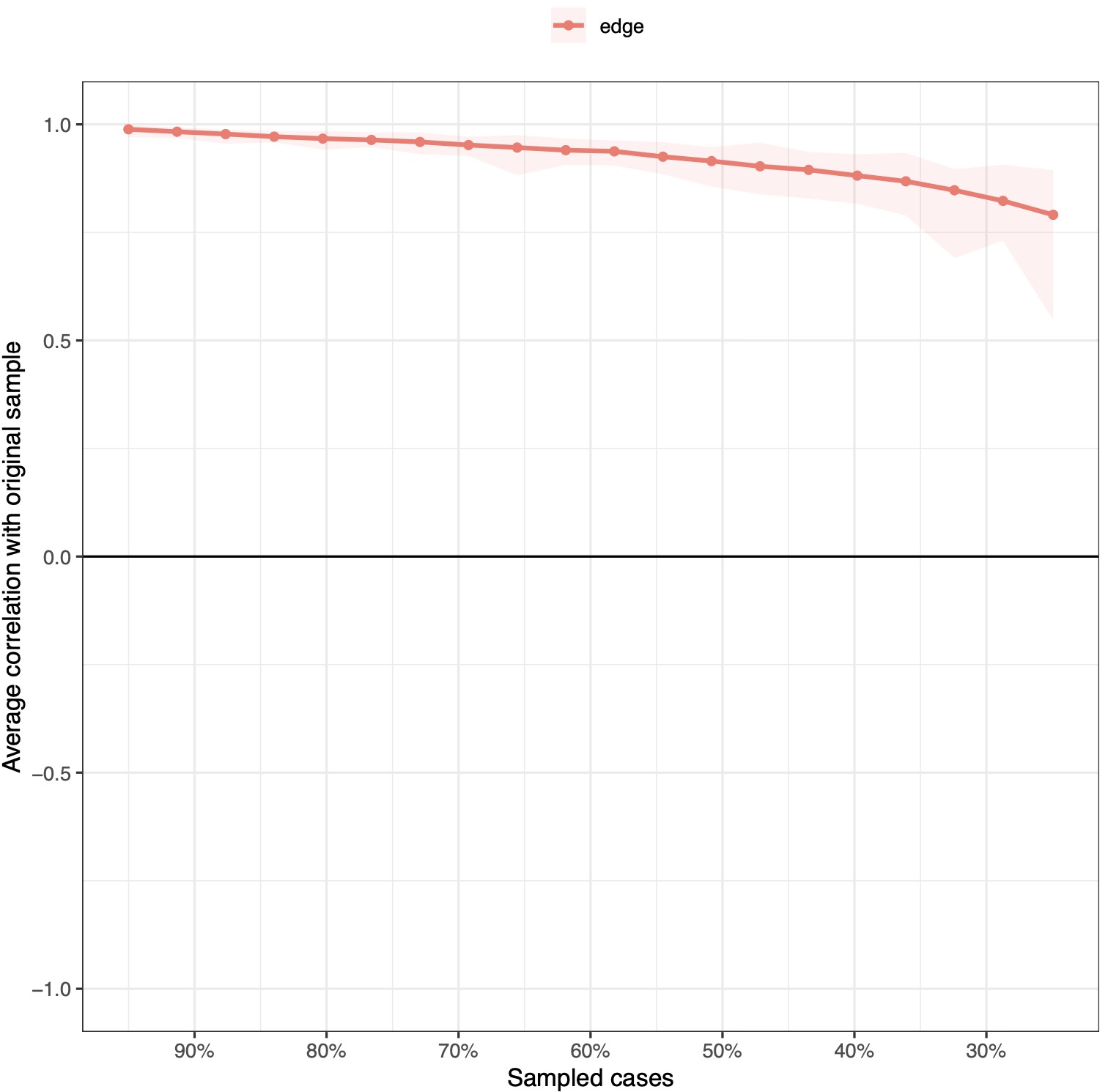

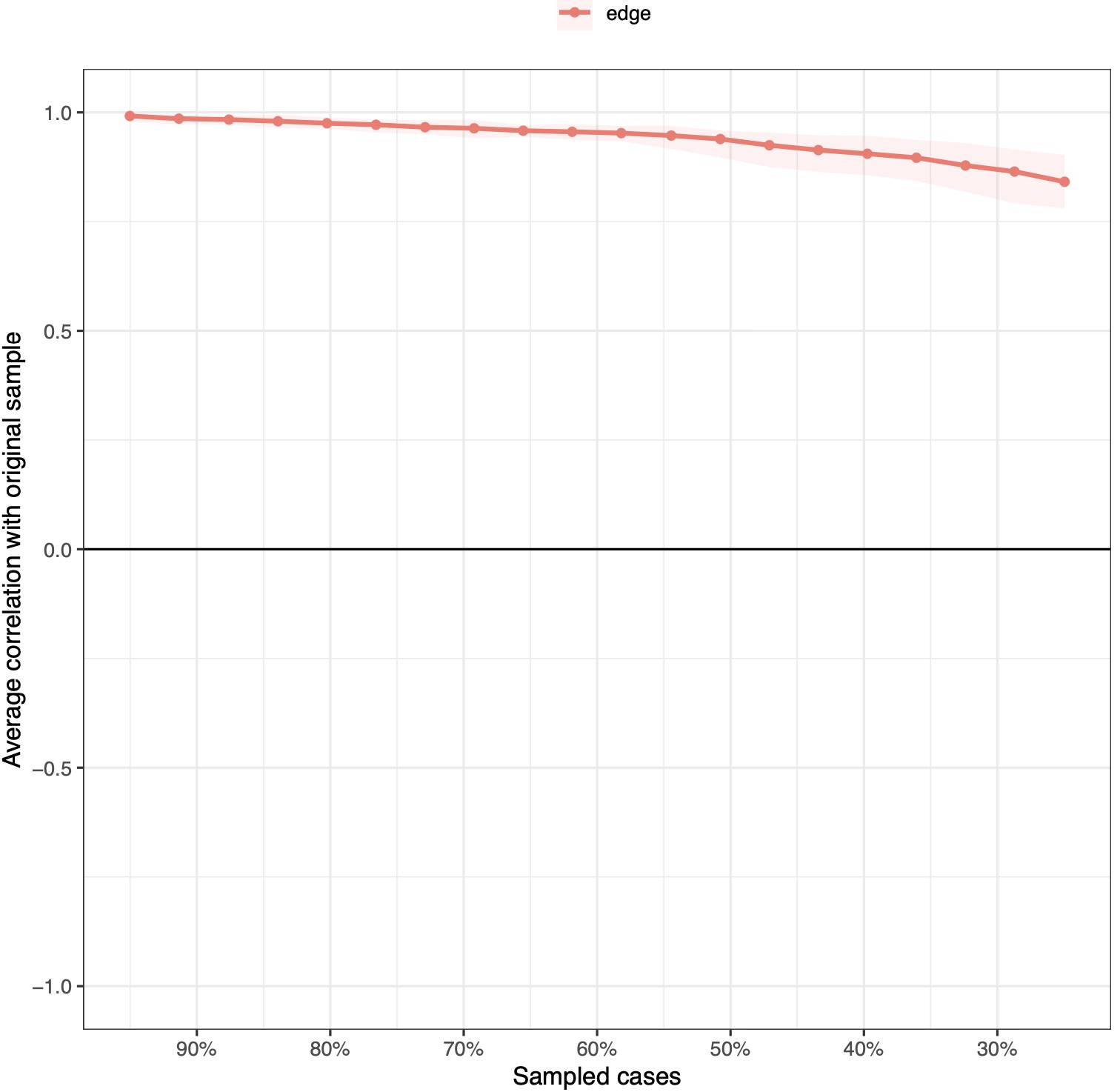
**

**Figure S1: Stability estimations of edges using the case-drop bootstrapping method of healthy profile transition group (left, n = 842) and deteriorating profile transition group (right, n = 1306)**


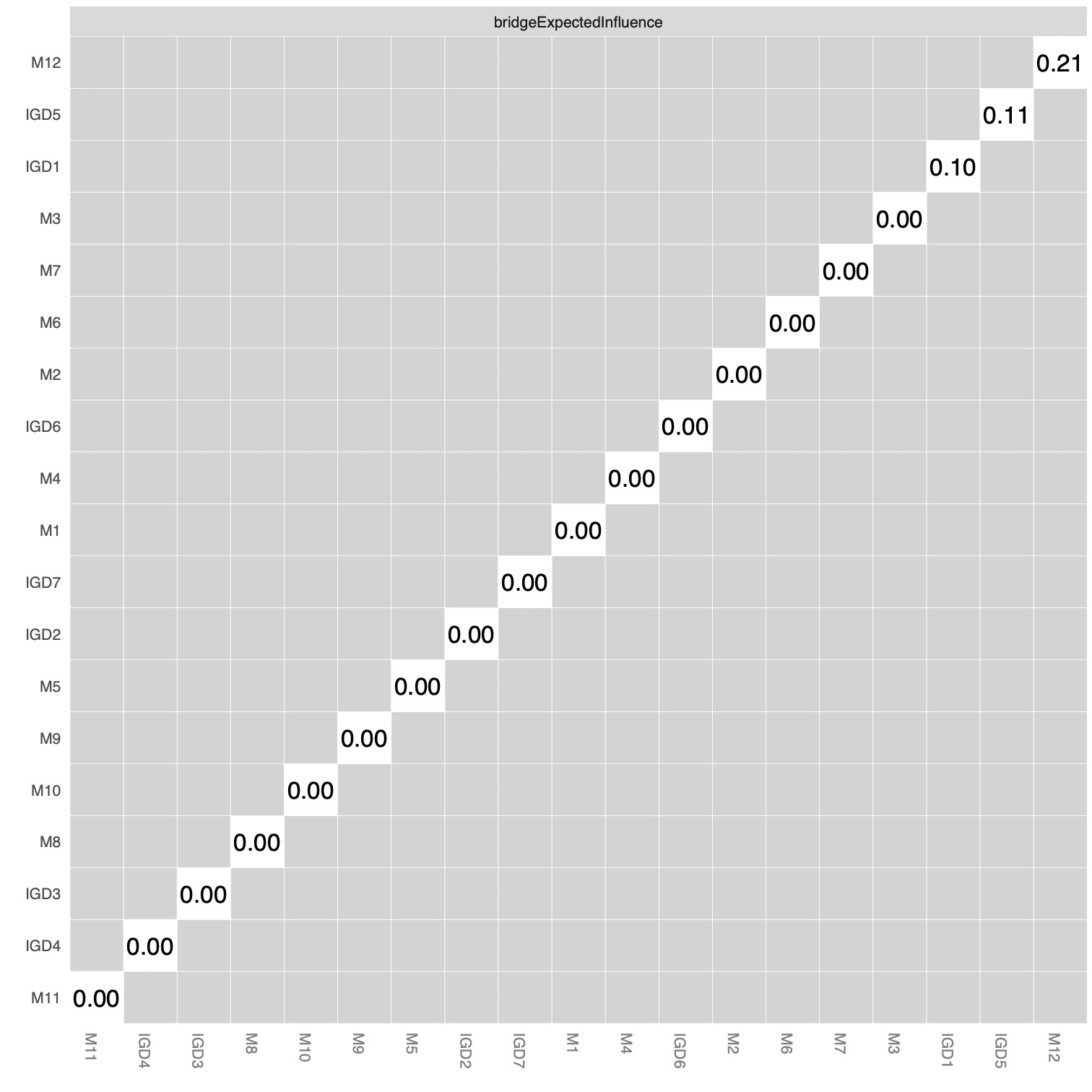

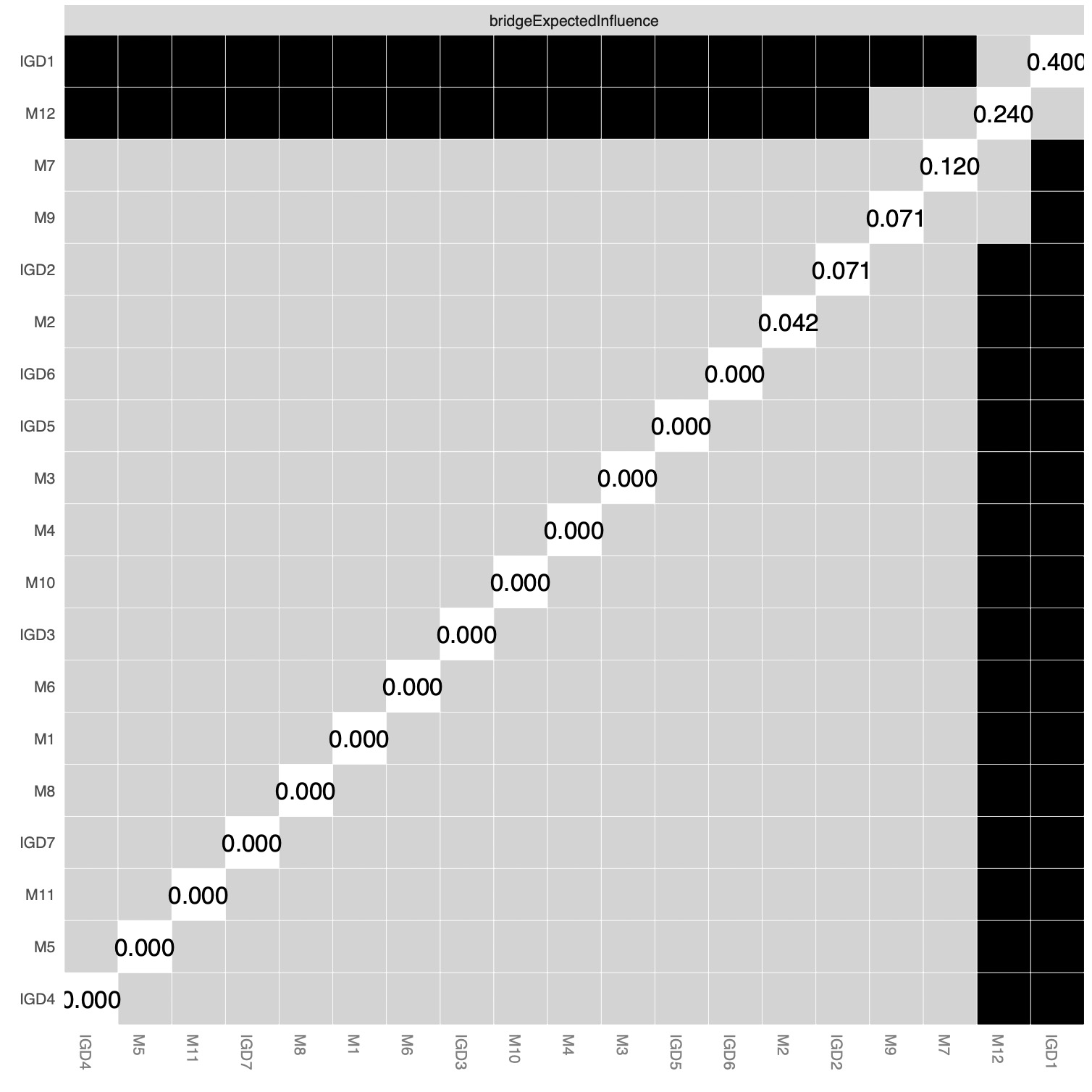


**Figure S2: The difference test results of bridge expected influence using the non-parametric bootstrapping method in healthy profile transition group (left, n = 842) and deteriorating profile transition group (right, n = 1306)**

**
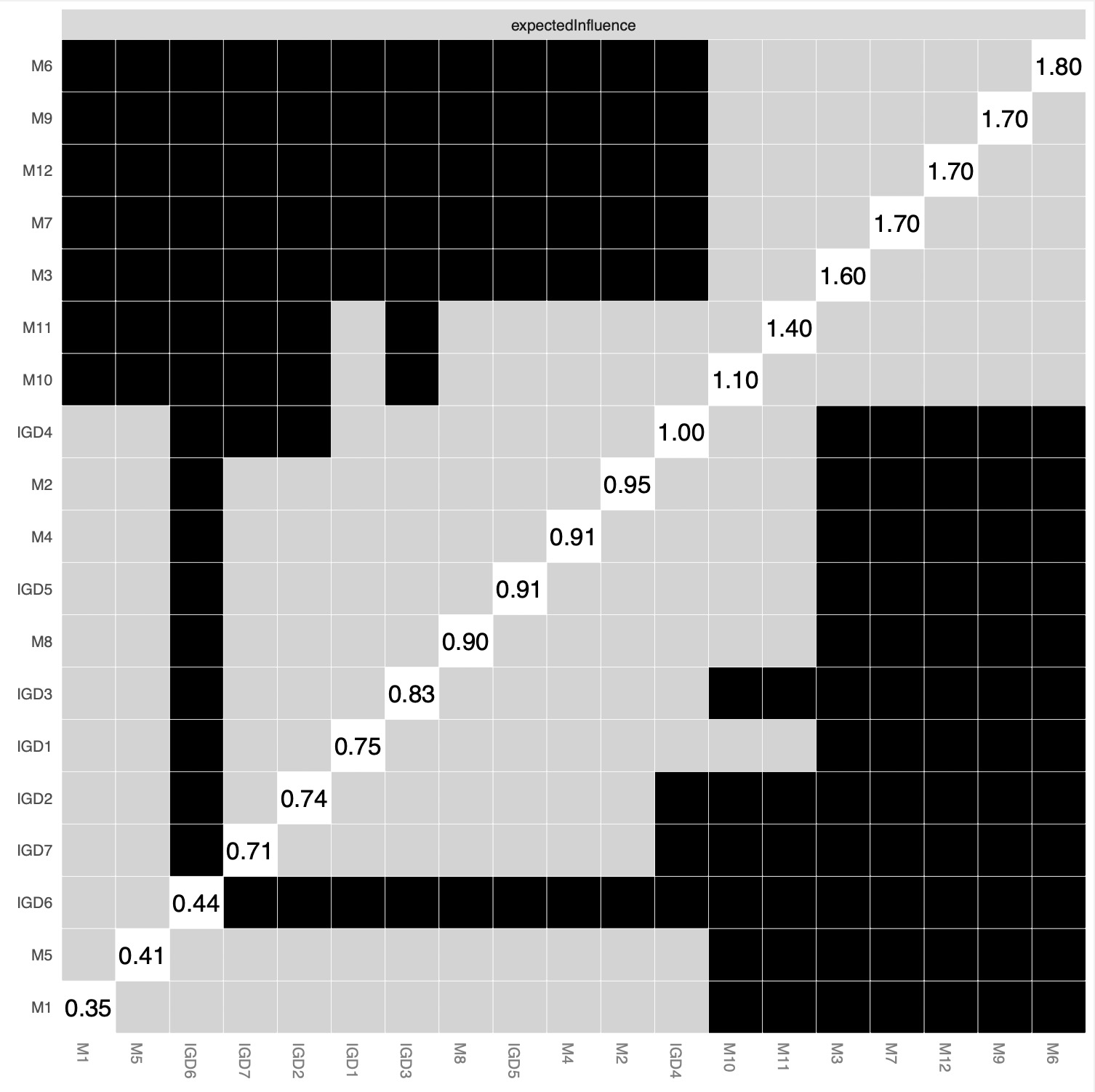
** **
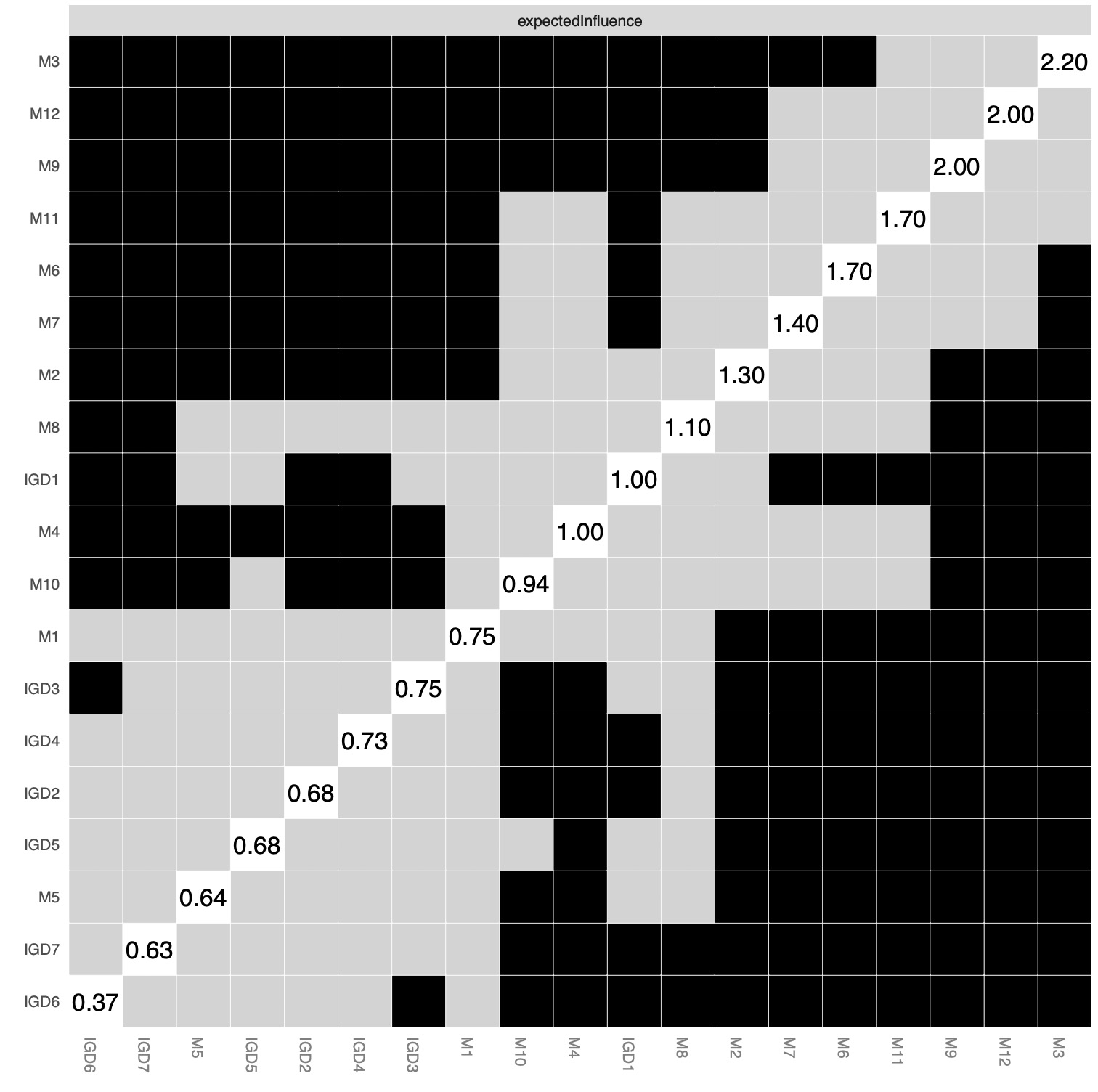
**

**Figure S3: The difference test results of expected influence using the non-parametric bootstrapping method in healthy profile transition group (left, n = 842) and deteriorating profile transition group (right, n = 1306)**
